# Resistance of *Fritillaria imperialis* to freezing stress through gene expression, osmotic adjustment and antioxidants

**DOI:** 10.1038/s41598-020-63006-7

**Published:** 2020-06-26

**Authors:** Shokoofeh Hajihashemi, Marian Brestic, Marco Landi, Milan Skalicky

**Affiliations:** 1 Plant Biology Department, Faculty of Science, Behbahan Khatam Alanbia University of Technology, Khuzestan, Iran; 2 Department of Plant Physiology, Faculty of Agrobiology and Food Resources, Slovak University of Agriculture, 94976 Nitra, Slovakia; 3 Department of Botany and Plant Physiology, Faculty of Agrobiology, Food, and Natural Resources, Czech University of Life Sciences, 16500 Prague, Czech Republic; 4 Department of Agriculture, Food and Environment, University of Pisa, Pisa, Italy; 5 Interdepartmental Research Center Nutrafood “Nutraceuticals and Food for Health”, University of Pisa, Pisa, Italy; 6 CIRSEC, Centre for Climatic Change Impact, University of Pisa, Via del Borghetto 80, I-56124 Pisa, Italy

**Keywords:** Ecology, Physiology, Plant sciences

## Abstract

Plant survival in response to freezing stress depends on the efficient activation of tolerance mechanisms. *Fritillaria imperialis* exposure to freezing stress enhanced signalling molecules Ca^2+^ and H_2_O_2_ along with overexpression of Ca^2+^ signalling proteins (Ca^2+^ dependent protein kinases, CPK), followed by upregulation of *NHX1* (Na^+^/H^+^ antiporter), *LEA* (late embryogenesis abundant proteins) and *P5CS* (1-pyrroline-5-carboxylate synthetase). Overexpression of *OsCNGC6* was responsible for high accumulation Ca^2+^, Na^+^ and K^+^. The NHX1 gene product transported Na^+^ to vacuoles and increased cytosolic K^+^ content to re-establish ionic homeostasis under stress conditions. The reduced water potential of leaves was due to high accumulation of osmolytes and ions. No changes were observed in relative water content of leaves, which might be correlated with overexpression of the *LEA* gene, which protects against dehydration. High accumulation of H_2_O_2_ under freezing stress was responsible for activation of antioxidant systems involving SOD, phenols, anthocyanins, catalase and ascorbate peroxidase. Photosynthesis, suppressed in freezing-stressed plants, returned to normal levels after termination of freezing stress. Taken together, our findings suggest that Fritillaria efficiently tolerated freezing stress through induction of signalling mechanisms and overexpression of cold stress-responsive genes, and prevention of cold-induced water stress, oxidative stress and photosynthetic damage.

## Introduction

*Fritillaria imperialis* is a perennial plant in the Liliaceae family native to Iran with beneficial medicinal qualities and ornamental importance. The orange-red downward facing flowers in combination with a crown of glossy green leaves at the top of stem make Fritillaria a very attractive horticultural species. One of the main habitats of wild Fritillaria in Iran is the protected area of Golestan-Kuh with an area of about 950 hectares and height of 3631 m. It is located 15 kilometres northeast of Khansar city, Isfahan Province which is subject to cold and snowy winters. Fritillaria require a period of cool temperatures to encourage flowering and its blooming happens in April at optimal temperatures of 18 to 20 °C. Fritillaria with other species of rangeland plants, on the southern slopes of Golestan-Kuh, are valuable natural cover vegetation, which attracts thousands of tourists every spring to visit this area^1–3^. *F. imperialis*, despite its tropical origin, is tolerant of cold stress, especially at the bulb stage^4,5^.

Cold stress is an abiotic stress that plants experience during extended exposure to freezing temperatures. A frost is especially harmful in late spring or early fall when plants are in an active growth stage^6^. Each plant has an optimum temperature range for its growth and development and conditions that are optimal for one plant, may be stressful for another^4,6^. Exposure to cold stress induced signalling mechanisms through an increase in cytosolic Ca^2+^ ^7^. The increase in cytosolic Ca^2+^ can be triggered through activation of cyclic nucleotide-gated ion channels (CNGCs) in response to cold stress^8^. Calcium-dependent protein kinases (CPKs) sense alterations in cytosolic Ca^2+^ level, and crosstalk with downstream signalling molecules including hormones, mitogen-activated protein kinases (MPKs) and reactive oxygen species (ROS), results in acclimation to the cold^7,9^. CPKs participates in signal transduction through the phosphorylation of MPKs and NADPH oxidase and thereby promote the production of ROS in response to environmental stress^7^. The increased generation of H_2_O_2_ in response to cold stress is accompanied by up-regulation of CPKs^7^.

Plants exposed to cold stress experience various changes in physiological and biochemical processes. Cold stress causes changes in photosynthesis, the levels of enzymatic and non-enzymatic antioxidants, and functions of cell membranes^10,11^. At low temperatures, cold-tolerant plants modify their homeostasis to achieve freezing tolerance^12^. The adjustment of metabolism in response to cold stress is mainly linked to activation of tolerance systems. Many metabolites are thought to function as osmolytes to regulate cellular water levels and reduce dehydration. This beneficial solute behaviour allows them to stabilize enzymes, membranes and other cellular components. Such stress-responsive metabolites include soluble sugars, amino acids, organic acids, and lipids^12–15^.

As one of the main plant processes, photosynthesis is very sensitive to temperature stress^14,16^. Recently, the measurement of chlorophyll (Chl) fluorescence has been used as a rapid tool for assessing function of the photosynthetic apparatus in response to environmental stress. Determination of F_v_/F_m_ (ratio of variable fluorescence to maximum fluorescence) allows the rapid detection of the degree of cold-stress damage to the photosynthetic system, PSII^14,17,18^. A linear correlation has been revealed between F_v_/F_m_ and quantum yield of photosynthesis. The F_v_/F_m_ ratio shows the quantum efficiency of PS II, while the absorbance performance index (PI_ABS_) indicates the efficiency of both PSs I and II. The quantification of net photosynthesis (P_N_), intercellular CO_2_ concentration (C_i_), and water use efficiency (WUE) provides additional information about the effects of stress on photosynthesis^17,18^.

Cold stress can also result in enhanced production of reactive oxygen species (ROS), but plant cells are well equipped with antioxidant systems able to scavenge free radicals, peroxides and other ROS. Reducing the production of ROS, as well as highly efficient ROS scavenging constitute effective strategies for coping with environmental trauma^12,13,15,19^. The antioxidant capacity depends on the activity of antioxidant enzymes in different cellular compartments as well as non-enzymatic antioxidants such as glutathione. Maintaining membrane and organelle integrity is closely correlated with ROS scavenging capacity and is thought to be a particular challenge under cold stress^6,20,21^.

The *LEA* gene encodes the late embryogenesis abundant (LEA) proteins, which are extremely hydrophilic and intrinsically disordered polypeptides that remain soluble after boiling and freezing. These features have led researchers to attribute a protective role to LEA proteins during cellular dehydration, temperature stress and salinity conditions (Kobayashi *et al*., 2004; Liu *et al*., 2014). It was reported that upregulation of the *LEA* gene increased the cold tolerance of *Camellia sinensis*^22^, *Nicotiana tabacum*^23^ and *Triticum aestivum*^24^. The *NHX*1 gene encodes a vacuolar Na^+^/H^+^ antiporter with a critical role in regulating salt and cold-stress tolerance through intercellular Na^+^ compartmentalization^25^. Overexpression of the *NHX*1 gene resulted in transport of Na^+^ from the cytosol to vacuoles and enhanced cytosolic K^+^ levels to re-establish ion homeostasis in response to environmental stress^26,27^, which improved cold tolerance of sweet potato^25^ and *Arabidopsis thaliana*^28^. Therefore, in this study the expression of *NHX1* and *LEA* genes in Fritillaria plants exposed to freezing stress were investigated.

In recent years, the ecosystem of Golestan-Kuh became vulnerable to cold stress because of temperature variations and unseasonable snowfall in spring. In order to determine the physiological traits that increase freezing tolerance by reducing injurious effects of low temperature, analysis of cold-resistant plants exposed to chilling in their natural habitat is a promising approach. Every plant species has an optimum temperature for growth and development, and many annual flowering plants are extremely sensitive to cold. *F. imperialis* is a good candidate for studying cold stress, however, since it can tolerate low temperature in habitats where most plants cannot survive^2,4,19^. Unraveling the physiological mechanism of *F. imperialis* for freezing tolerance may provide the information we need to genetically engineer cold tolerance into crop plants. To the best of our knowledge, the physiological and biochemical traits that enable *Fritillaria* to withstand freezing have so far not been investigated. The measurement of physiological parameters of plants under unfavourable conditions in the field is valuable, because it enhances our understanding about plant conservation under stress. The present study aimed at elucidating physiological mechanisms that allow *F. imperialis* to cope with cold stress in its natural habitat. For this purpose, we analyzed the basic parameters of photosynthesis (Chl fluorescence, F_v_/F_m_), the performance index of PSI and PSII (PI_ABS_), intercellular CO_2_ (C_i_), net-photosynthesis (P_N_), water use efficiency (WUE), chlorophylls), metabolites (proteins, proline, water soluble sugars (WSS), total reducing sugar (TRS), phenols and anthocyanins), the antioxidant system (total antioxidant power (FRAP), superoxide dismutase (SOD), catalase (CAT), ascorbate peroxidase (APX), and polyphenol oxidase (PPO)), lipid peroxidation, ions (K^+^, Na^+^ and Ca^2+^), and expression of *CNGC*, *CPK*, *NHX1* and *LEA* genes in *Fritillaria* under freezing stress.

## Materials and Methods

### Plant stress and sampling

On the first day of April 2016, an unseasonal storm in the mountains at Golestan-Kuh brought snow and a fifteen-degree decrease in minimum temperature from 10 °C to −5 °C. On April 2^nd^, the low temperature reached −10 °C, but by the following day the minimum had risen to a more normal 8 °C. An April 3^rd^, examination of the Fritillaria habitat showed no trace of snow around the Fritillaria plants on the hillside location (33°09′19.2″N 50°24′12.4″E), while plants in the highlands locations (33°08′46.1″N 50°23′42.7″E) were covered with snow. By April 8th, however, the air temperature had risen to 14 °C and all the snow in the mountain highlands Fritillaria habitat had melted. The unseasonable drop in air temperature in the mountain highlands resulted in freezing stress on the Fritillaria, which was evidenced by the leaf colour changing from green to dark red. Plants on the hillside where there was no snow showed no change in leaf colour. To study the physiological changes in response to freezing stress, plants were harvested on April 8^th^ from the mountain highlands and hillside areas. As the weather got warmer, the dark red colour of the leaves returned to green. The second plant collection happened one week later, when there was a freeze on April 15^th^. By April 22^nd^ the air temperature had returned to normal at 18 °C, the Fritillaria recovered and the leaves all turned green again. The third plant harvesting happened on April 22^nd^. Leaves were collected from at least five plants at each location. For photosynthetic parameters measurement, the same plants were analyzed before harvesting.

### Analysis of photosynthetic and fluorescence parameters

The maximum quantum yield of PS II (F_v_/F_m_) and the performance index of both PS I and II (PI_ABS_) were measured using a portable chlorophyll fluorimeter (Pocket PEA, Hansatech, England). The Chl fluorescence was measured on ten fully expanded leaves from five plants. To measure F_v_/F_m_ and PI_ABS_, the leaves were acclimated in the dark from night to early morning, then illuminated with saturating light (3500 μmol m^−2^ s^−1^). Net-photosynthesis (P_N_), water use efficiency (WUE) and intercellular CO_2_ (C_i_) were measured at 10 AM in the same plants using a portable photosynthesis meter system (KR8700; Korea Tech Inc., Korea)^17^, then, a portable chlorophyll meter (CCM – 200 plus Chlorophyll Content Meter, Thailand) was used to measure total Chls in the same plants. After measurement of photosynthetic parameters, the leaves were harvested for analysis of physiological and biochemical activity.

### Analysis of physiological parameters

Water potential (ψ_w_) represents the force that causes water to move through the plant. The leaf ψ_w_ was measured by the method of Martìnez *et al*.^29^. The relative water content (RWC %) of leaves was determined based on the following formula:

$$\begin{array}{c}{\rm{RWC}}( \% )=[({\rm{FW}}-{\rm{DW}})/{\rm{TW}}-{\rm{DW}})]\ast 100,\,{\rm{with}}\,{\rm{FW}}:{\rm{fresh}}\,{\rm{weight}}\,({\rm{g}}),\\ {\rm{TW}}:{\rm{turgor}}\,{\rm{weight}}({\rm{g}}),\,{\rm{DW}}:{\rm{dry}}\,{\rm{weight}}({\rm{g}}).\end{array}$$

To measure carbohydrates, oven-dried leaves were ground to a fine powder and extracted with boiling water for 15 min. Water soluble carbohydrates (WSCs) were determined using the phenol-sulfuric acid assay^30^. Reducing sugars (RS) were measured based on the method of Somogyi^31^. Free proline was extracted from fresh leaves with 3% (w/v) aqueous sulfosalicylic acid and measured according to the method of Bates, Waldren^32^. Enzymes and other proteins were extracted from fresh leaves by macerating them in sodium phosphate buffer (50 mM, pH 7.8) containing 5 mM magnesium sulfate, 4 mM dithiothreitol, 1.0 mM EDTA, and 2% (w/v) polyvinyl polypyrrolidone at 4 °C^33^. Protein concentration was determined by Bradford^34^ assay. The activity of superoxide dismutase (SOD) was measured according to Beauchamp and Fridovich^35^, catalase (CAT) as described by Aebi^36^, ascorbate peroxidase (APX) according to Asada^37^, and polyphenol oxidase (PPO) as described by Flurkey and Jen^38^. To measure H_2_O_2_, fresh leaves were extracted with trichloroacetic acid^33^ and assayed according to Velikova, Yordanov^39^. Phenols were extracted from fresh leaves with ethanol and measured using Folin’s reagent^40^. An acidic methanol solution was used for extracting anthocyanins, which were measured with the Wagner^41^ method. The quantification of total antioxidant power (FRAP) of fresh leaves was performed by the Szôllôsi and Varga^42^ method. To measure malondialdehyde (MDA), leaves were extracted with trichloroacetic acid. Lipid peroxidation was quantified based on the MDA measurement according to the Heath and Packer^43^ assay. The fine powder obtained from grinding oven-dried leaves was digested with HClO_4_ at 200 °C to measure Ca^2+^, K^+^ and Na^+^ ions^44^.

### Expression of the cyclic nucleotide-gated ion channel (*OsCNGC6*), the Ca^2+^-dependent protein kinases (*OsCPK17*), the Na^+^/H^+^ antiporter (*NHX*_*1*_) and *LEA* genes

Total RNA was extracted from leaves using the plant RNA mini kit (Invitrogen), according to the manufacturer’s instructions. The cDNA synthesis was done as already described by Hajihashemi, Geuns^45^. The primers used for both target and housekeeping genes were: *OsCNGC6*: forward (5′TTCTGCGCACAAAGCTCAAT3′)

reverse (5′GCTAAACTTCAGGGTGCTCCT3′)

*OsCPK17*: forward (5′AATAAGCCCAAGGTGAGG3′)

reverse (5′CGAGTCCTCCTTATGGTTG3′)

Na^+^/H^+^ antiporter: forward (5′CCACTTCCGATCATGCTTCT3′)

reverse (5′AAGAATGCCACTCAGATAGG3′)

*LEA*: forward (5′ATAAGGACACCACCACCACT3′)

reverse (5′TTAAAGCTCAGGATCTCGGC3′)

*P5CS*: forward (5′CATCCCTGTTTCTCTCCACC3′)

reverse (5′CCATCTCGCGTACATCAACC3′)

actin: forward (5′GCTCTGCCCGTTGCTCTGATGAT3′)

reverse (5′CCTTGGATGTGGTAGCCGTTTCT3′)

Real-time quantitative PCR, based on the fluorescence emitted by the amplification products in the presence of SYBR green, was done to quantify the expression of target genes^45^.

### Statistical analysis

The means of F_v_/F_m_, PI_ABS_, P_N_, WUE, C_i_, and Chls were obtained from ten leaves from five plants. The means of water potential, relative water content, carbohydrates, proline, protein, phenol, anthocyanins, MDA, FRAP, activity of CAT, APX, SOD and PPO enzymes, Ca^2+^, Na^+^, K^+^ and expression of *OsCNGC6*, *OsCPK17*, *NHX*_*1*_, *P5CS* and *LEA* genes are the mean of four values for each treatment. Data were analyzed using SPSS statistical (version 24) software. Treatment means were subjected to ANOVA and significant differences between data were measured by Duncan’s multiple range test (*p* ≤ 0.05). To show significant differences, the statistically analyzed data are shown with superscripted letters after the numbers in figures.

## Results

**Figure 1:**
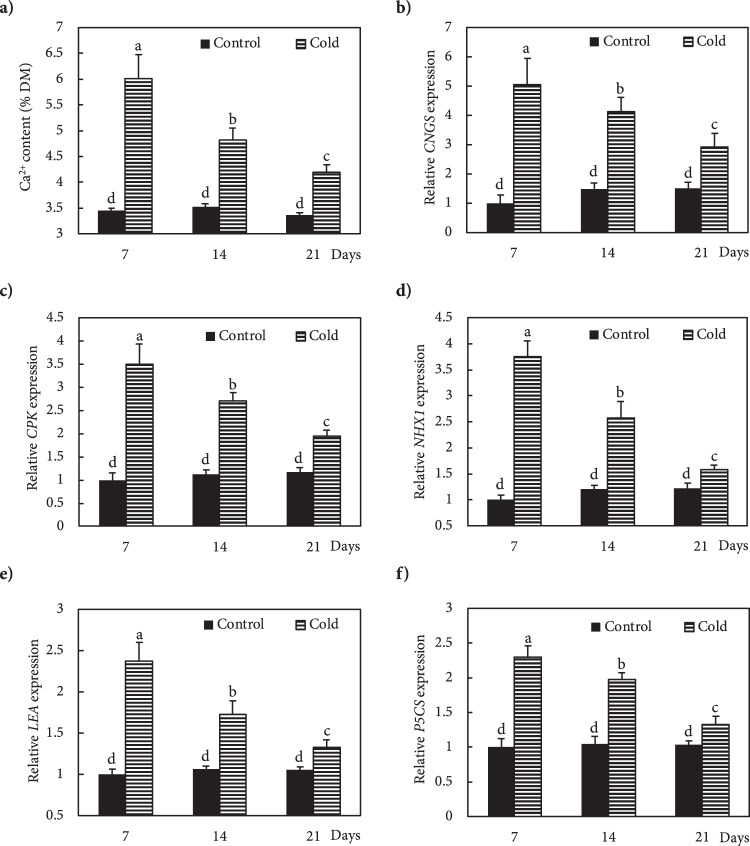
Effect of freezing stress on relative expression of (**a**) Ca^2+^ content, (**b**) *OsCNGC6* gene (encodes a cyclic nucleotide-gated ion channel) *OsCNGC6* gene (encodes a cyclic nucleotide-gated ion channel) and (**c**) *OsCPK17* (encodes a Ca^2+^-dependent protein kinase), (**d**) *NHX1* gene (encodes a vacuolar Na^+^/H^+^ antiporter), (**e**) *LEA* (encodes late embryogenesis abundant proteins) and (**f**) *P5CS* gene (encodes 1-pyrroline-5-carboxylate synthetase). The measurements were conducted in Fritillaria plants at days 7, 14 and 21 days after snowing (7, 14 and 21 DAS). Data are the means and standard errors of four replicates (n = 4). Data with different letters reveal statistically significant differences among the treatments according to a Duncan’s multiple range test (*p* < 0.05).

**Figure 2:**
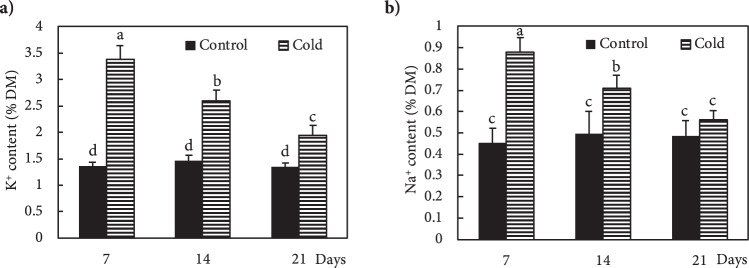
Effect of freezing stress on (**a**) K^+^ content and (**b**) Na^+^ content. The measurements were conducted in Fritillaria plants at days 7, 14 and 21 days after snowing (7, 14 and 21 DAS). Data are the means and standard errors of four replicates (n = 4). Data with different letters reveal statistically significant differences among the treatments according to a Duncan’s multiple range test (*p* < 0.05).

**Figure 3:**
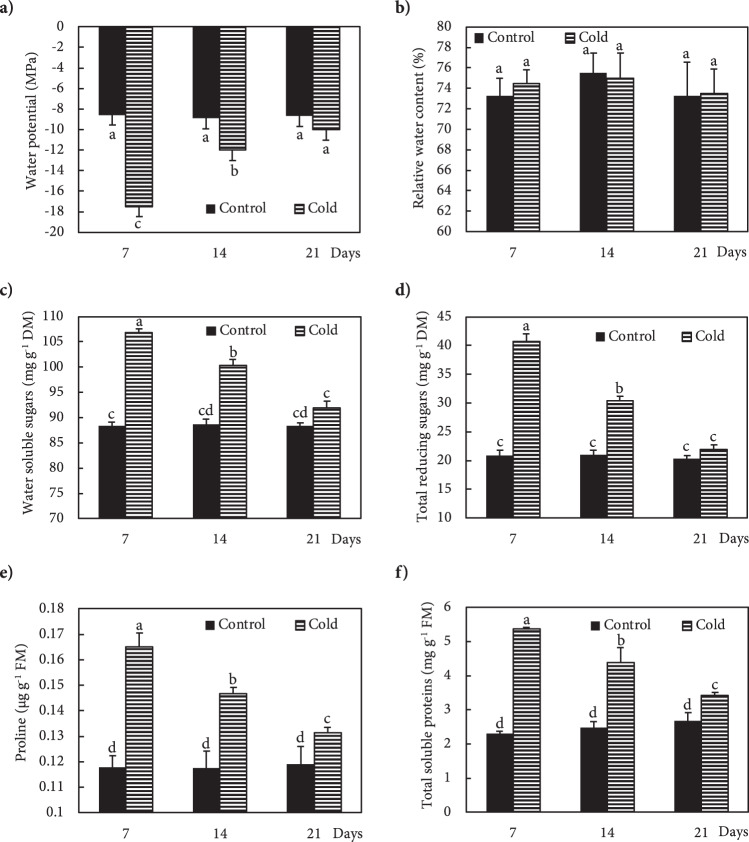
Effect of freezing stress on the contents of (**a**) water potential, (**b**) relative water content, (**c**) water soluble carbohydrates, (**d**) total reducing sugars, (**e**) proline and (**f**) total soluble proteins in leaves. The measurements were conducted in Fritillaria plants at days 7, 14 and 21 days after snowing (7, 14 and 21 DAS). Data are the means and standard errors of four replicates (n = 4). Data with different letters reveal statistically significant differences among the treatments according to a Duncan’s multiple range test (*p* < 0.05).

**Figure 4:**
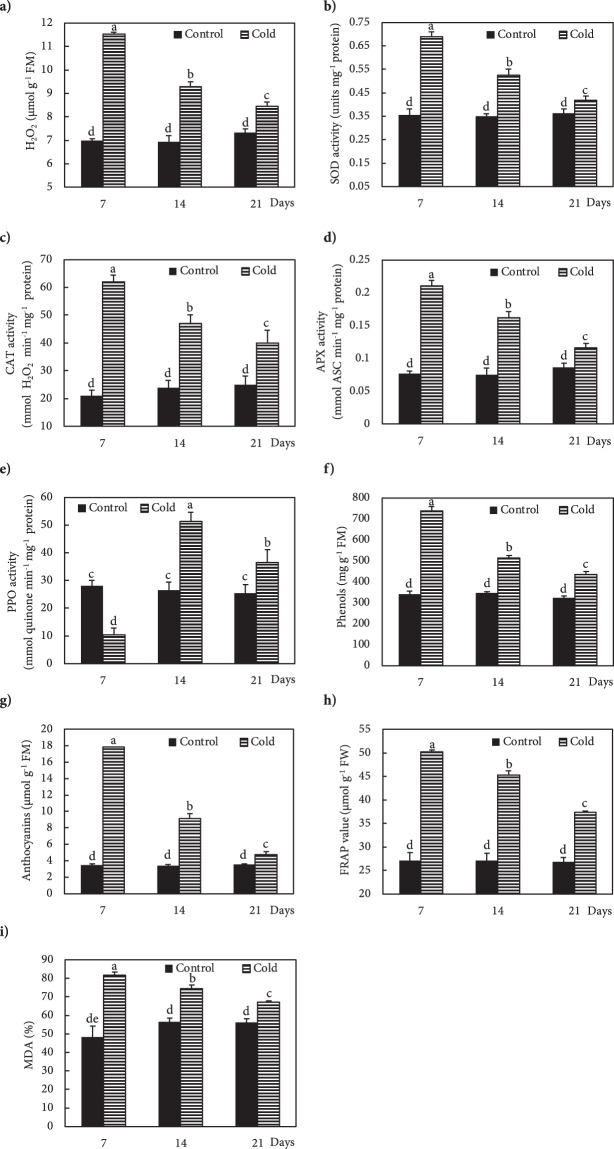
Effect of freezing stress on (**a**) H_2_O_2_, (**b**) super oxidase dismutase activity (SOD), (**c**) catalase activity (CAT), (**d**) ascorbate peroxidase activity (APX), (**e**) polyphenol oxidase activity (PPO), (**f**) phenols, (**g**) anthocyanins, (**h**) total antioxidant power (FRAP), and (**i**) malondialdehyde (MDA) in leaves. The measurements were conducted in Fritillaria plants at days 7, 14 and 21 days after snowing (7, 14 and 21 DAS). Data are the means and standard errors of four replicates (n = 4). Data with different letters reveal statistically significant differences among the treatments according to a Duncan’s multiple range test (*p* < 0.05).

**Figure 5:**
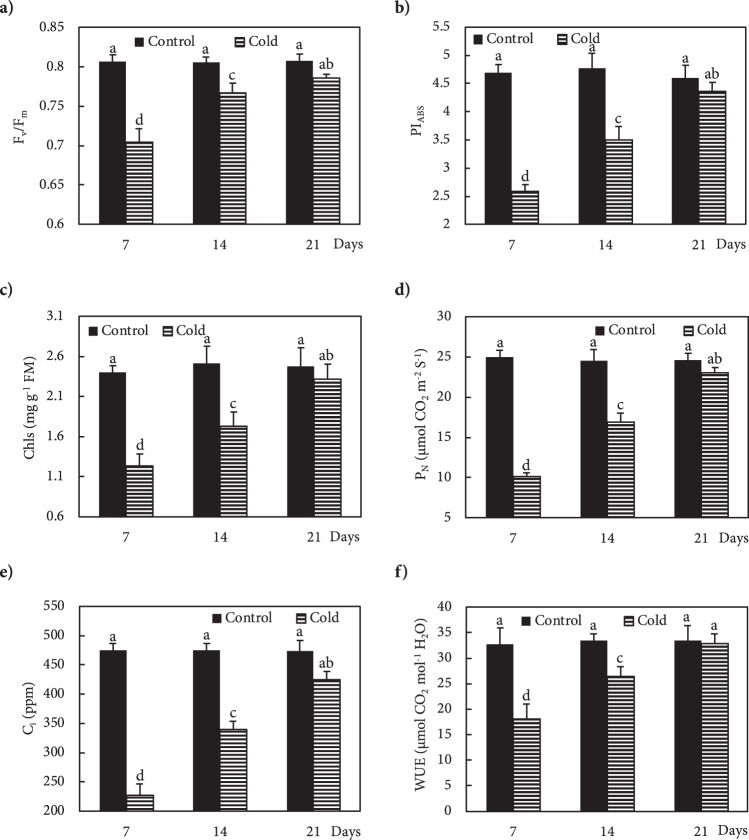
Effect of freezing stress on photosynthetic features of (**a**) maximum quantum yield of photosystem II (F_v_/F_m_), (**b**) performance indices (PI_ABS_), (**c**) total chlorophylls (Chls), (**d**) net photosynthesis (P_N_), (**e**) intercellular CO_2_ (C_i_), and (**f**) water use efficiency (WUE). The measurements were conducted in Fritillaria plants at days 7, 14 and 21 days after snowing (7, 14 and 21 DAS). Data are the means and standard errors of four replicates (n = 4). Data with different letters reveal statistically significant differences among the treatments according to a Duncan’s multiple range test (*p* < 0.05).

Freezing stress increased the Ca^2+^ level in the leaves of Fritillaria by 74, 36 and 25% at 7, 14 and 21 DAS (days after snow), respectively, as compared to non-stressed plants (Fig. 1a). Under freezing stress conditions, the increase in Ca^2+^ was accompanied by an increase in *OsCNGC6* expression by 405, 180 and 95% at 7, 14 and 21 DAS, respectively, relative to non-stressed plants (Fig. 1b). Expression of the *OsCPK17* gene in the stressed plants increased by 250, 140 and 66% at 7, 14 and 21 DAS, respectively, compared to non-stressed plants (Fig. 1c). The expression of the *NHX1* gene stressed plants increased by 275, 115 and 129% at 7, 14 and 21 DAS, respectively, compared to non-stressed plants (Fig. 1d). Under freezing stress conditions, the expression of the *LEA* gene increased by 137, 62 and 25% at 7, 14 and 21 DAS, respectively, relative to controls (Fig. 1e). In response to freezing stress, the expression of *P5CS* gene increased by 130, 87 and 27% at 7, 14 and 21 DAS, respectively, compared to non-stressed plants (Fig. 1f). Freezing stress induced a large increase in K^+^ in the leaves by 149, 78 and 43% at 7, 14 and 21 DAS, respectively, compared to non-stressed plants (Fig. 2a). In addition, plants exposed to freezing showed an increase in Na^+^ content of 94, 43 and 15% at 7, 14 and 21 DAS, respectively, relative to non-stressed plants (Fig. 2b). The Ψ_w_ of leaves subjected to freezing showed a significant reduction of 104% at day 7 and 35% at day14 after snowing (DAS), while no significant change occurred at 21 DAS, compared to controls (Fig. 3a). The RWC of leaves from stressed plants showed no significant changes at 7, 14 and 21 DAS, compared to non-stressed plants (Fig. 3b). WSC and RS in stressed plants increased by 20 and 95% at 7 DAS, and 13 and 44% at 14 DAS, respectively, versus non-stressed plants (Fig. 3c,d). Proline content in stressed plants increased 40, 24 and 10% at 7, 14 and 21 DAS, respectively, relative to non-stressed plants (Fig. 3e). Freezing stress increased protein levels by 132, 77 and 12% at 7, 14 and 21 DAS, respectively, over levels in non-stressed plants (Fig. 3f). Under non-stressed conditions, the H_2_O_2_ concentration in leaves showed no significant changes at 7, 14 and 21 DAS, while freezing stress significantly increased H_2_O_2_ content: 65, 33 and 15% at 7, 14 and 21 DAS, respectively (Fig. 4a). After freezing stress, the SOD activity of leaves increased by 93, 50 and 15% at 7, 14 and 21 DAS, respectively over that of corresponding non-stressed plants (Fig. 4b). Freezing stress increased the activity of CAT by 194, 96 and 60% at 7, 14 and 21 DAS, respectively (Fig. 4c). The activity of APX increased in stressed plants by 175, 115 and 34% at 7, 14 and 21 DAS, respectively (Fig. 4d). Under freezing stress, the PPO activity decreased by 63% at 7 DAS, but increased by 93 and 44% at 14 and 21 DAS, respectively (Fig. 4e). Under non-stressed conditions, the level of total phenolics in leaves was not altered at 7, 14 and 21 DAS, while it increased by 117, 48 and 35% at 7, 14 and 21 DAS, respectively (Fig. 4f). Freezing stress increased the level of anthocyanins by 419, 167 and 34% at 7, 14 and 21 DAS, respectively (Fig. 4g). Under non-stressed conditions, the FRAP value of leaves showed no significant differences, while freezing increased FRAP by 86, 67 and 39%, at 7, 14 and 21 DAS, respectively (Fig. 4h). Under non-stressed conditions, the level of MDA did not change significantly at 7, 14 and 21 DAS, respectively, while exposure of plants to freezing stress increased the MDA level by 70, 32 and 19% at 7, 14 and 21 DAS, respectively (Fig. 4i). Freezing stress decreased the F_v_/F_m_ ratio by13, 5 and 3% at 7, 14 and 21 DAS, respectively (Fig. 5a). Stress induced a decrease in PI_ABS_ by 45, 27 and 5%, at 7, 14 and 21 DAS, respectively (Fig. 5b). The Chls levels of stressed plants decreased by 49, 31 and 6% at 7, 14 and 21 DAS, respectively (Fig. 5c). Under freezing stress conditions, the P_N_ level decreased by 59, 31 and 6% at 7, 14 and 21 DAS, respectively (Fig. 5d). In comparison with non-stressed plants, stressed plants showed a decrease in C_i_ level of 52, 29 and 10% at 7, 14 and 21 DAS, respectively (Fig. 5e). Under freezing-stressed conditions, the WUE value decreased by 45, 21 and 2% at 7, 14 and 21 DAS, respectively (Fig. 5f).

**Figure 6:**
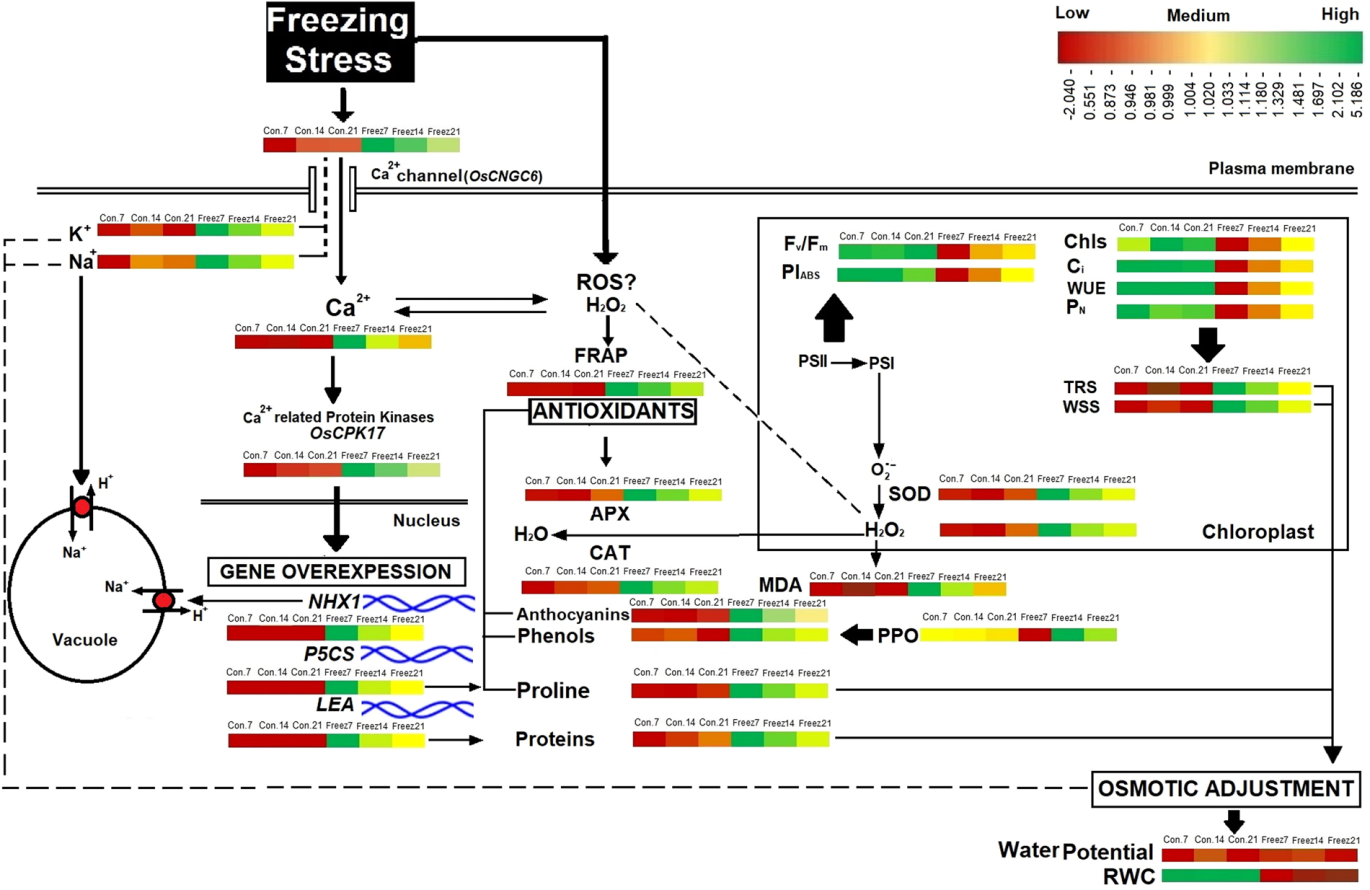
The pattern of changes in the metabolites of Fritillaria in response to freezing stress. The values are expressed as the changes happened in the parameters as compared to their values in the control plants at 7 days after snowing and then plotted as a heat map with the color scale of red (lowest value) to green (highest value). Data are from control (Con.) and freezing-stressed (Freez.) plants at days 7, 14 and 21 days after snowing (7, 14 and 21 DAS). Anth, anthocyanins; APX, ascorbate peroxidase; CAT, catalase; Chls, total chlorophylls, C_i_, intercellular CO_2_; *CNGC*, cyclic nucleotide-gated ion channel; *CPK*, Ca^2+^ dependent protein kinases; FRAP, total antioxidant power; F_v_/F_m_, maximum quantum yield of photosystem II; *LEA* gene, late embryogenesis abundant; MDA, malondealdehyde; *NHX1* gene, a vacuolar Na^+^/H^+^ antiporter; PI_ABS_, performance indices; P_N_, net photosynthesis; *P5CS* gene, PPO, polyphenol oxidase; RWC, relative water content; SOD, super oxide dismutase; TRS, total reducing sugars; WSS, water soluble sugars; WUE, water use efficiency; Ψw, water potential.

The pattern of responses of the different variables to freezing stress in Fritillaria 7, 14 and 21 DAS is depicted in Fig. 6 using heat map data. The heat map shows that the variables of anthocyanins, CAT, APX, proteins, phenols, TRS, SOD, FRAP, MDA, H_2_O_2_, proline, WSS, Ca^2+^, Na^+^, K^+^, and expression of *OSCNGC6*, *OsCPK17*, *NHX1* and *LEA* genes did not change significantly in control plants from 7 DAS to 21 DAS (*red*), while their values significantly increased in response to freezing stress with the highest level at 7 DAS (*dark green*). In addition, their values showed a downward trend to values similar to those in the control plants as daytime temperatures increased. Freezing stress had no effect on RWC, which is shown in red in the heat map. The photosynthetic function was significantly suppressed in the stressed plants. The F_v_/F_m_, WUE, PI_ABS_, Chls, C_i_, and P_N_ (*green*) showed higher values in control plants than in plants exposed to freezing stress (*red*). The values of photosynthetic variables increased to a level similar to control plants by 21 DAS, which demonstrates the ability of the photosynthetic process in Fritillaria to recover after freezing stress. The PPO response to freezing stress showed a negative relationship between this enzyme and phenol content. Freezing stress induced a significant reduction in Ψw (*red*).

## Discussion

Efficient tolerance of plants to unfavourable growth conditions is a basic prerequisite for survival under environmental stress. Temperature is one of the main factors, which determines the geographic distribution of organisms, and the tolerance of plants to cold stress depends on efficiency of their cellular signal transduction mechanisms. Messenger molecules, like H_2_O_2_ and Ca^2+^, and protein kinases, like CPKs, are involved in cold-stress signalling pathways that enhance a plant’s cold resistance by increasing the transcription of stress-responsive genes and antioxidant capacity^46^. Cold stress rapidly leads to an influx of Ca^2+^ through Ca^2+^channels, like CNGCs, into the cytosol^8^. The observed increase in Ca^2+^ levels in the leaves along with higher expression of the *OsCNGC6* gene suggests the existence of active signalling pathways in Fritillaria that induce expression of cold-tolerance genes against freezing stress. The *OsCNGC6* is one of the most highly expressed *CNGCs* in response to cold stress^47^. The CNGCs-mediated Ca^2+^ influx results in Ca^2+^ binding to CPKs^8^, which is similar to the observed increase in Ca^2+^ concentration followed by over-expression of *OsCPK17* in Fritillaria exposed to freezing. Almadanim, Alexandre^48^ suggested a correlation between overexpression of *OsCPK17*, sugar metabolism and osmotic regulation in cold-stressed rice, which is in agreement with the results of the present study. The overexpression of CPK in *Vitis amurensis* increased the expression of downstream stress-responsive genes, such as *NHX1*, *LEA*, *P5CS* and *CNGC* under cold and drought stress^49^, which confirms our results here. H_2_O_2_ triggers and amplifies the signalling pathway to induce expression of cold-tolerance genes and antioxidant systems against the effects of low temperature exposure^46^. Freezing stress enhanced H_2_O_2_ accumulation in Fritillaria, which Si, Wang^46^ suggested served as a signal inducing a response to cold stress. Lv, Li^7^ reported that CPK-induced H_2_O_2_ accumulation promoted adaptation to cold stress, which was evidenced here in the response of Fritillaria to low temperature stress.

Plants apply different adaptive mechanisms to withstand and survive stress. Strategies for dealing with environmental stress include two main types: avoidance that prevents the stress factor from modifying plant functioning, and tolerance in which physiological mechanisms are activated or modified to either resist stress or repair the damage. Thus, a plant’s resistance level to a given stress in its natural habitat depends on its ability to activate resistance mechanisms and/or its adaptations for avoidance^6^. Therefore, there is a need to develop greater knowledge about the tolerance mechanisms of stress-tolerant plants in order to engineer crops with high resistance to environmental stress utilizing biotechnology. The sudden occurrence of a period of lower than usual temperatures in a plant’s normal niche, can induce cold shock that causes injury or death^6^. Some species of Fritillaria are endangered and they all have great commercial importance as ornamentals and potential sources for therapeutic compounds^1–3^; thus, the mechanism of its resistance to sudden freezing conditions in its niche, and the physiological and biochemical evidence for this were evaluated and discussed in this study.

The exposure of Fritillaria to freezing stress, due to an unseasonable snowfall in its natural habitat of Golestan-Kuh in April and a large drop in air temperature, promoted a significant reduction in Ψ_W_ of leaves. Low temperatures can lead to dehydration by impeding water uptake, which causes a reduction in water potential^50^. Monitoring the changes of Ψ_W_ showed the highest reduction at 7 DAS, after which the process recovered to the normal levels seen in control plants at 21 DAS. The recovery of Ψ_W_ from a low level at 7 DAS to normal at 21 DAS seemed to correlate with the accumulation of protective solutes such as sugars, proline and proteins in response to freezing stress. In contrast to Ψ_W_, no reduction in the RWC of leaves was observed in cold-stressed Fritillaria, which suggested activation of tolerance mechanisms in this species. Plants survive harsh winters by overcoming the two major causes of freezing injury, cell dehydration and membrane damage^51^. The formation of intracellular ice crystals can kill cells by damaging their membranes. The ice crystals formed in the intercellular spaces may also cause dehydration, due to their very low water potential, which leads to cell damage^51,52^. Plants resist ice formation at freezing temperatures by the generation of high levels of osmolytic solutes, which inhibit cellular water in protoplasm from freezing by reducing its freezing point. Accordingly, water remains in the liquid form even at temperatures below 0 °C^53,54^. Plants regulate their osmotic potential through the accumulation of compatible osmolytes such as sugars, proline and proteins that help to maintain normal hydration and prevent the formation of ice crystals^13,15,50,51^. Sugar accumulation plays an important role in acquisition of cold tolerance. Our results confirmed that there was a reverse correlation between reduction of Ψ_W_ and accumulation of WSS and TRS under freezing conditions. The accumulation rate of TRS in plants exposed to temperatures below freezing was higher than for the WSS rate, suggesting that TRS had a greater influence on promoting the reduction of Ψ_W_. In the current study, the proline content increased in stressed plants, which was consistent with the results of^52,54^ who reported that cold stress increased the proline content. The increase in proline was accompanied by a significant increase in expression of the *P5CS* gene, and upregulation of proline biosynthetic genes like *P5CS* after cold stress has been reported^55^. A large accumulation of proteins was observed in Fritillaria exposed to freezing stress, which might be correlated with overexpression of *LEA* to maintain appropriate water potential, and thus sustain RWC. At 7 DAS, the highest levels of sugars, proline and proteins were accompanied by the largest reduction of Ψ_W_. The increase of the temperature from 7 DAS to 21 DAS induced a gradual increase in Ψ_W_, along with a gradual reduction in osmolytes to a level similar to that in control plants. The LEA proteins have a protective ability against dehydration conditions^56^, and Sasaki, Christov^56^ reported the boiling solubility, hyper-hydrophilicity, stress inducibility and stress-protecting ability of LEA proteins induced during cold acclimation in *Triticum aestivum*. Thus, the overexpression of LEA in Fritillaria suggested their involvement in improving its cold tolerance through different mechanisms.

It is also known that plants able to avoid cold-induced water stress and maintain normal hydration of cells in response to low temperature have high cold tolerance^57,58^. Although the functional mechanism of up-regulation of *NHX1* attributed to freezing tolerance remain unknown, the observed overexpression of *NHX1* in Fritillaria exposed to below freezing temperatures followed by high accumulation of Na^+^ and K^+^ might explain the plant’s ability to maintain cellular water status with no change in RWC. These results are similar to a previous report on *Arabidopsis* about the role of *NHX1* in intracellular K^+^/Na^+^ ionic homeostasis and maintaining water status of cells subjected to dehydration stress^59^. Under dehydration conditions, Na^+^ as an osmolyte, can maintain an osmotic potential to drive water into cells. Its compartmentation in the vacuole by the Na^+^/H^+^ antiporter is a critical process to prevent its toxic effect in cytosol^59^. Taken together, the observed high Na^+^ and K^+^ content together with the up-regulation of *NHX1* could be the reasons that Fritillaria avoid freezing -induced water stress. Dong, Wang^60^ reported that overexpression of *NHX* in tobacco was accompanied by an increase in antioxidant enzyme activity and lower levels of ROS, which is in agreement with our results on freezing-stressed Fritillaria.

The CNGCs family members are known to be involved in Na^+^, K^+^ and Ca^2+^ uptake^8^ and the accumulation of Na^+^ and K^+^ in cold-stressed Fritillaria might be explained by overexpression of *OsCNGC6*. Freezing stress causes an imbalance in the generation and scavenging of ROS in plants, resulting in oxidative stress-induced deterioration of membrane lipids, which can lead to cell death^21,52,54^. In this study, the ROS levels were quantified through measurement of H_2_O_2_ and malondialdehyde (MDA). Both H_2_O_2_ and MDA increased under freezing stress in Fritillaria plants, with the highest levels at 7 DAS. As the DAS were extended and the air temperature increased, the H_2_O_2_ and MDA levels gradually decreased to become similar to those in control plants by 21 DAS. Therefore, we concluded that Fritillaria had the ability to both scavenge ROS and recover from freezing stress-induced injuries to cell membranes. The configuration ability of LEA proteins when bound to lipids, suggested their involvement in membrane stabilization under environmental stress^56^. Therefore, the reduction in MDA in stressed Fritillaria from 7 to 21 DAS might also be attributable to up-regulation of the *LEA* gene. Campo, Baldrich^61^ reported an overexpression of *LEA* genes involved in the dehydration response of rice to drought and salt stress, which might suggest a correlation between *OsCPK17* and *LEA* genes to protect Fritillaria against cold-induced dehydration.

Alleviation of cold stress-induced oxidative damage is a key mechanism in cold stress tolerance, and plants evolved antioxidant defence systems, including the mobilization of non-enzymatic and enzymatic antioxidants to overcome ROS-induced oxidative stress^10,21,62,63^. SOD is the first enzyme to perform detoxification of ROS by converting the superoxide radical into a H_2_O_2_ molecule, which CAT and APX enzymes then immediately convert into H_2_O^6,33,63^. Freezing stress significantly increased the activity of SOD at 7 DAS, which was accompanied by accumulation of H_2_O_2_. The SOD activity declined progressively from 7 to 21 DAS along with the concentration of H_2_O_2_. Results of current investigation also revealed a significant increase in activities of the antioxidant enzymes CAT and APX in freezing stress-exposed plants, linked to high level of H_2_O_2_. Accordingly, it is likely that the enhanced activities of CAT and APX enzymes could effectively eliminate the overproduced H_2_O_2_. Even though, the levels of H_2_O_2_ and MDA at 21 DAS were higher than control plants, the reduction in H_2_O_2_ and MDA accumulation in freezing-stressed plants from 65% to 15% and 70% to 19%, respectively, from 7 DAS to 21 DAS reflected the effective enzymatic antioxidant system of Fritillaria against freezing stress and its role as protector against cold stress-induced oxidative stress^12,21,41^. The antioxidant ability of glucose might be a result of increasing the NADPH concentration through activation of the pentose phosphate pathway. The NADH molecule is one of the main cofactors of ROS-scavenging enzymes^64,65^, and the elevated ROS detoxification activity in cold-stressed Fritillaria might contribute to the high accumulation of NADPH. Carbohydrates also play an important role in the stabilization of cell membranes through insertion between the polar groups of lipids, which can decrease membrane permeability^66,67^. For that reason, the elevation in sugar content induced by freezing might explain its important role in preventing membrane damage and acquisition of freezing tolerance.

In parallel with the increased activity of antioxidant enzymes, the phenol content also increased in freezing-stressed Fritillaria, which contributed to protecting them from cold-induced oxidative damage because of the antioxidant activity of phenols^21^. It has been reported that tolerant plants exposed to cold stress accumulated high levels of phenolic compounds^21,68^. The findings of this study confirmed the previous reports where cold stress increased phenolic compounds and reduced the adverse effects of oxidative stress^6,21,54^. Freezing stress suppressed the activity of PPO at 7 DAS, which was accompanied by the highest level of phenolic compounds observed at 7 DAS. These data demonstrated an antiparallel correlation among the total phenolic compounds and PPO activity in Fritillaria exposed to freezing. Furthermore, these results showed that the increased PPO activity from 7 to 21 DAS was accompanied by a decrease in total phenolic compounds from 7 to 21 DAS. In addition, the levels of anthocyanins increased following freezing stress in Fritillaria, with the highest and lowest levels at 7 and 21 DAS, respectively. Anthocyanins are a class of flavonoid with antioxidant properties, which play an important role in ROS scavenging and increasing the cold tolerance of plants^69–71^. High levels of phenols and anthocyanins are responsible for cold stress-induced pigmentation in the leaves, which increases the antioxidant capacity of plants^68,70,71^. Cold stress increased the expression of anthocyanin synthase genes in *Brassica rapa* followed by accumulation of high levels of anthocyanins, causing the leaves to appear dark red and enhancing cold tolerance^70^. Accordingly, the conversion of green Fritillaria leaves to dark red at the initial stages of freezing stress might be the result of an elevated accumulation of phenols and anthocyanins, which is supported by the Ahmed, Park^70^ report. Increased FRAP content in parallel with increased enzymatic and non-enzymatic antioxidant levels might be ascribed to decreased accumulations of H_2_O_2_ and MDA from 7 to 21 DAS. Several studies reported that increased-levels of sugars, anthocyanins, phenols, and APX and CAT activity could improve ROS scavenging in cold-stressed plants^6,12,21,41,64,67,68^. Taken together, the results of this investigation indicated that the improved freezing tolerance of Fritillaria might be due to its increased ability to prevent ROS-induced oxidative damage.

The reduction of photosynthetic rates is one of the problems caused by low temperatures, since the cold stress affects both electronic transport in thylakoids and carbon fixation^6,16,17,72^. In cold-tolerant plants, the suppression of photosynthesis can occur within a few hours after cold exposure, which then returns to previous photosynthetic values after the return to higher temperatures^6^. The reduction in photosynthetic pigments induced by cold stress can also contribute to the reduction in photosynthesis^6,17,73^. The negative effects of freezing stress on chlorophyll fluorescence and photosynthetic parameters including F_v_/F_m_, PI_ABS_, Chls, P_N_, C_i_ and WUE was reversed from 7 to 21 DAS, which might be attributable to antioxidant mechanisms and osmotic adjustment. An increase in chlorophyll fluorescence may occur in cold-tolerant plants after restoration of the optimum temperature, which indicates recovery of the photosynthetic process^6,73^. Contributing to our understanding of the effects of cold-stress on photosynthesis, several lines of evidence have suggested that tolerant plants are able to stimulate specific cellular processes, including activation of antioxidant systems and accumulation of osmoprotective metabolites^19,51,53,64^. The photosynthetic efficiency drops after freezing stress with its lowest level at 7 DAS, while increases in levels of WSS and TRS, with their highest levels at 7 DAS, provide evidence of their role in maintaining osmotic homeostasis. Cold stress suppresses the transport of sugars from sink to source to maintain osmotic balance and water status in cells to reduce damage (Hajihashemi *et al*., 2018). The increased levels of TRS and WSS concomitant with the reduced photosynthetic characteristics and chlorophylls in Fritillaria exposed to freezing temperatures could ensure the availability of sufficient resources for cold tolerance^41,64,74^. Feedback inhibition of photosynthetic process happens as a result of decreased sugar mobilization from source to sink^75^. Therefore, an increase in sugars in cold-stressed Fritillaria might be a result of the prevention of mobilization of sugars from source to sink. The accumulation of sugars in the leaves of Fritillaria as a ‘source’ might explain the suppression of photosynthesis at early stages of cold stress until the sugar level has decreased over time after the freezing event and the photosynthetic apparatus has been sufficiently reactivated to provide the required photosynthates. Taken together, the improved performance of the photosynthetic apparatus and Chls contents in Fritillaria, evidenced by increased levels of F_v_/F_m_, PI_ABS_, Chls, P_N_, C_i_ and WUE, has enhanced our understanding of how the photosynthesis process recovers to control levels after the freezing stress has passed.

Cold-induced signalling pathways were mediated by increased concentrations of signalling molecules, Ca^2+^ and H_2_O_2_, and overexpression of *OsCPK17* and *OsCNGC6* in freezing-stressed Fritillaria. The overexpression of *OsCNGC6* increased Ca^2+^influx followed by an increase in *OsCPK17* and subsequent overexpression of the downstream stress-response genes, *LEA*, *NHX1* and *P5CS*. The overexpression of stress tolerance genes, accumulation of osmolyte solutes, and induction of enzymatic and non-enzymatic antioxidants improved cell water status, removed ROS, and recovered normal photosynthetic processes in freezing-stressed plants. The restoration of sufficiency in photosynthetic function of Fritillaria was noticeably accompanied by the conversion of the dark red colour of freezing-stressed leaves to a normal green colour similar to that seen in control plants. In conclusion, the results of the present study demonstrate that Fritillaria has a very effective system of cold tolerance, although the participation of all cold acclimation genes in the process has not been deduced. Overall, the active signalling pathways, sufficient osmotic adjustment ability and antioxidant systems provide promising avenues for genetic engineering of cold-tolerance in crop plants as well as ornamentals.

## Data Availability

All data of manuscript are available.
